# Supplementary material for: CRISPRi-based screen of Autism Spectrum Disorder risk genes in microglia uncovers roles of ADNP in microglia endocytosis and synaptic pruning
Source: bioRxiv. 2024 Nov 17:2024.06.01.596962. Preprint. [Version 2] doi: 10.1101/2024.06.01.596962 (PMC11601228; doi:10.1101/2024.06.01.596962)
Supplement: Supplement 7 [file NIHPP2024.06.01.596962v2-supplement-7.pdf]

bioRxiv preprint doi: https://doi.org/10.1101/2024.06.01.596962; this version posted November 17, 2024. The copyright holder for this preprint (which was not certified by peer review) is the author/funder, who has granted bioRxiv a license to display the preprint in perpetuity. It is made available under aCC-BY 4.0 International license.

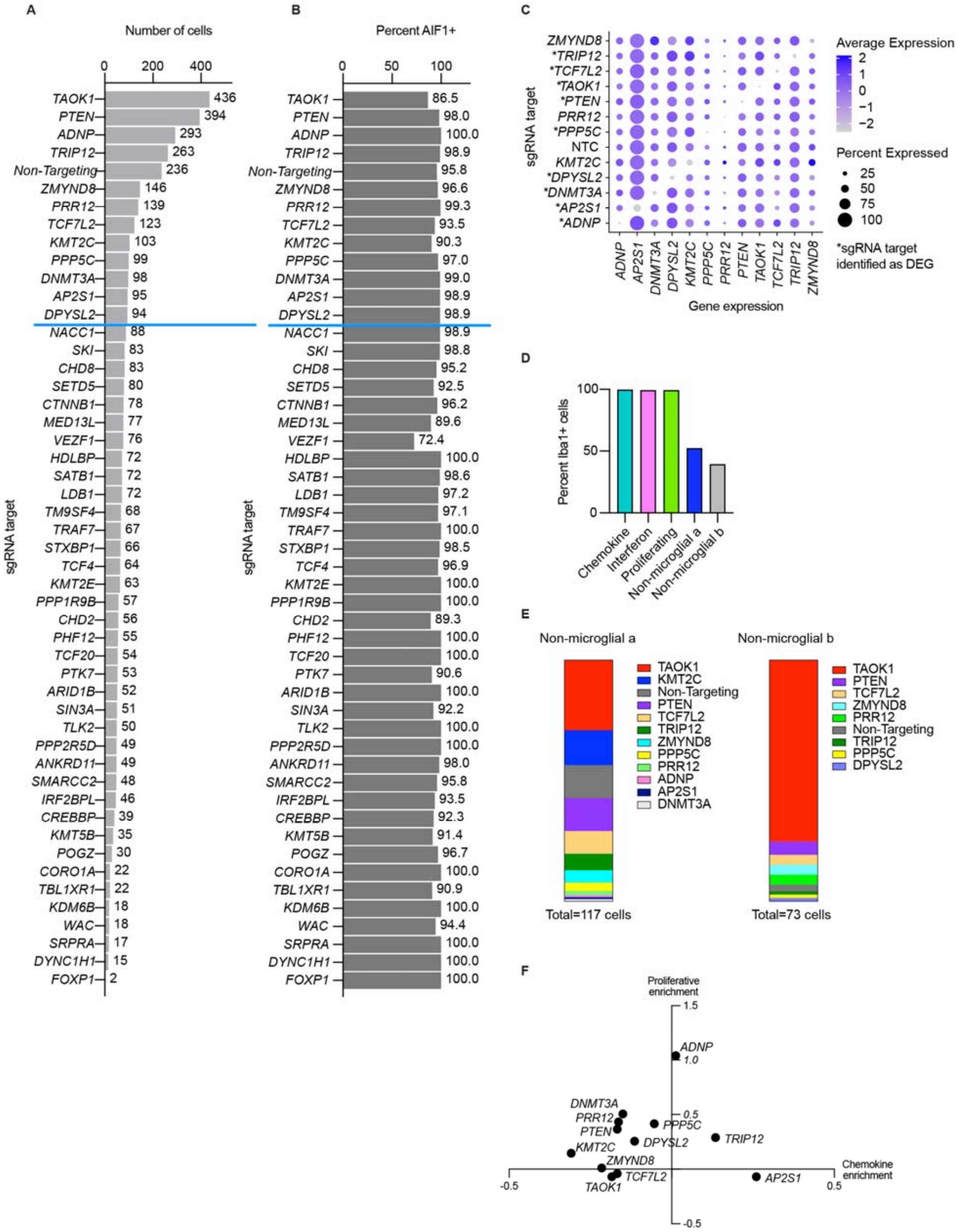

bioRxiv preprint doi: https://doi.org/10.1101/2024.06.01.596962; this version posted November 17, 2024. The copyright holder for this preprint (which was not certified by peer review) is the author/funder, who has granted bioRxiv a license to display the preprint in perpetuity. It is made available under aCC-BY 4.0 International license.

Supplemental Fig. 1: CROP-Seq quality control. A. Bar graph of the number of cells for each gene knockdown (KD). Gene KDs with greater than 90 cells were further studied. B. Bar graph of the percent Iba1 or *AIF1* positive cells for each gene KD. C. Dot plot displaying the efficacy of gene KDs (x axis) across 12 different gene KDs and non-targeting control sgRNAs (y axis). The color of the dots reflect the relative expression of the gene, and the size of the dots represent the percent of cells expressing the gene for a given gene KD. D. Percent *AIF1* (Iba1) positive cells in each cluster. E. Composition of non-microglial clusters a and b in terms of cells and their specific gene knockdown. F. Scatterplot showing the change in the proliferative and chemokine clusters relative to non-targeting controls.

bioRxiv preprint doi: https://doi.org/10.1101/2024.06.01.596962; this version posted November 17, 2024. The copyright holder for this preprint (which was not certified by peer review) is the author/funder, who has granted bioRxiv a license to display the preprint in perpetuity. It is made available under aCC-BY 4.0 International license.

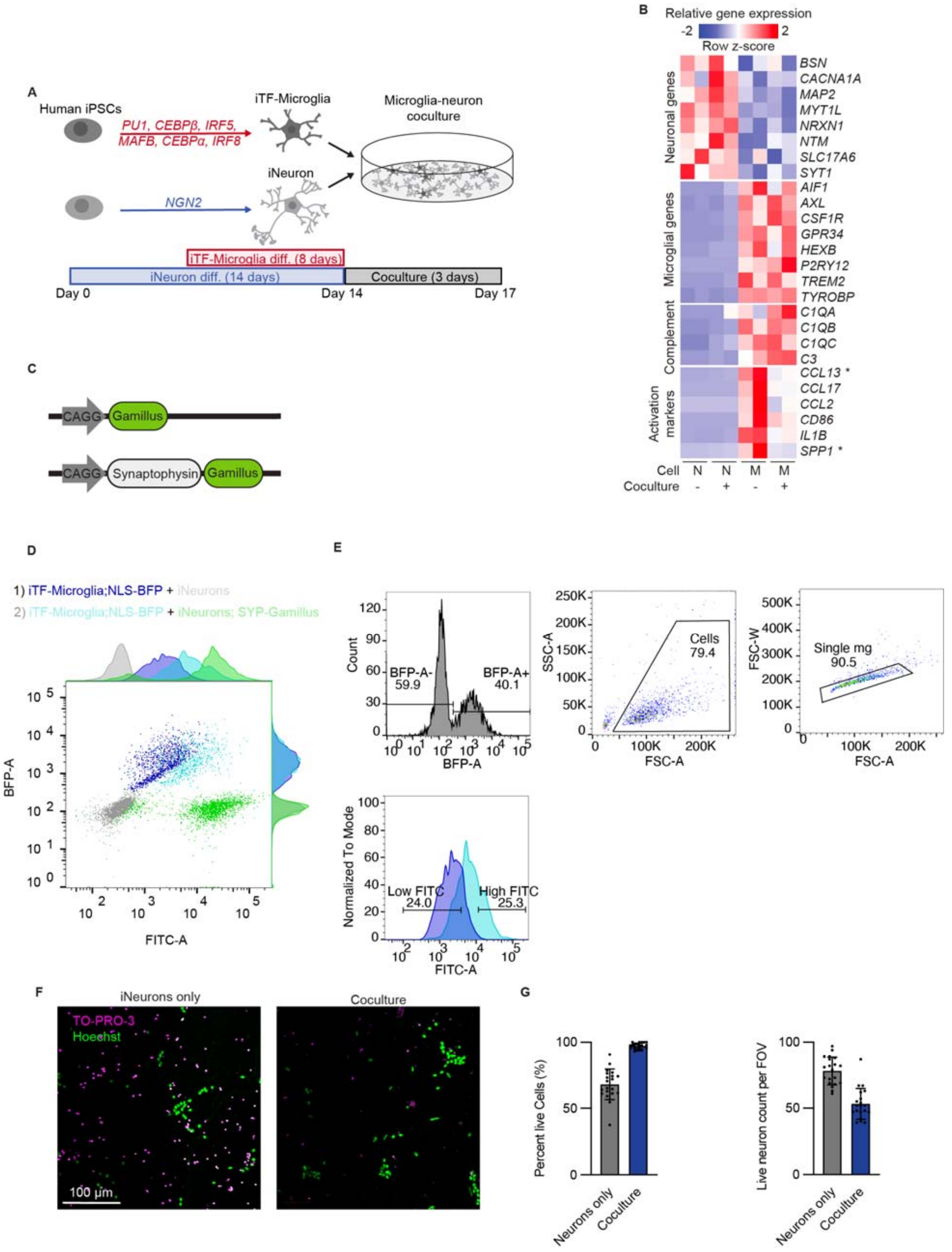

bioRxiv preprint doi: https://doi.org/10.1101/2024.06.01.596962; this version posted November 17, 2024. The copyright holder for this preprint (which was not certified by peer review) is the author/funder, who has granted bioRxiv a license to display the preprint in perpetuity. It is made available under aCC-BY 4.0 International license.

Supplemental Fig. 2: Characterization of cocultures of iPSC-derived neuron (iNeuron) and iPSC-derived microglia (iTF-Microglia) and synaptic material uptake. A. iTF-Microglia and iNeuron differentiation and coculture strategy. B. Heatmap showing relative gene expression from RNA sequencing of neuronal genes, microglial genes, complement components, and microglial activation markers by iTF-Microglia (M) and iNeurons (N) in monoculture and coculture preparations (N = 2 biological replicates). C. Gamillus expression constructs for cytosolic Gamillus (top) and synaptophysin linked to Gamillus (bottom). D. Representative scatterplot showing FITC and BFP channels from flow cytometry analysis of iTF-Microglia and neuron cocultures. Two coculture configurations are displayed: a coculture of iTF-Microglia with a nuclear blue fluorescent protein marker and iNeurons with no engineering and a coculture of of iTF-Microglia with a nuclear blue fluorescent protein marker and iNeurons expressing synaptophysin linked to a green fluorescent protein, Gamillus. E. Gating strategy for isolating iTF-Microglia from coculture and a comparison of the FITC shift in iTF-Microglia cocultured with iNeurons (dark blue) and iTF-Microglia cocultured with iNeurons expressing synaptophysin linked to a green fluorescent protein, Gamillus (light blue). F. Representative micrographs iNeuron monocultures (left) and iTF-Microglia and iNeuron coculture (right) stained with the dead cell indicator, TO-PRO-3 (magenta), and nuclear stain, Hoechst 33342 (green). G. (Left) Percent live cells based on nuclear TO-PRO-3 intensity (N = 4 fields of view (FOV) for 5 wells, bars represent mean +/- standard deviation). (Right) Total cell count per field of view based on nuclear Hoechst segmentations (N = 4 fields of view for 5 wells, bars represent mean +/- standard deviation).

bioRxiv preprint doi: https://doi.org/10.1101/2024.06.01.596962; this version posted November 17, 2024. The copyright holder for this preprint (which was not certified by peer review) is the author/funder, who has granted bioRxiv a license to display the preprint in perpetuity. It is made available under aCC-BY 4.0 International license.

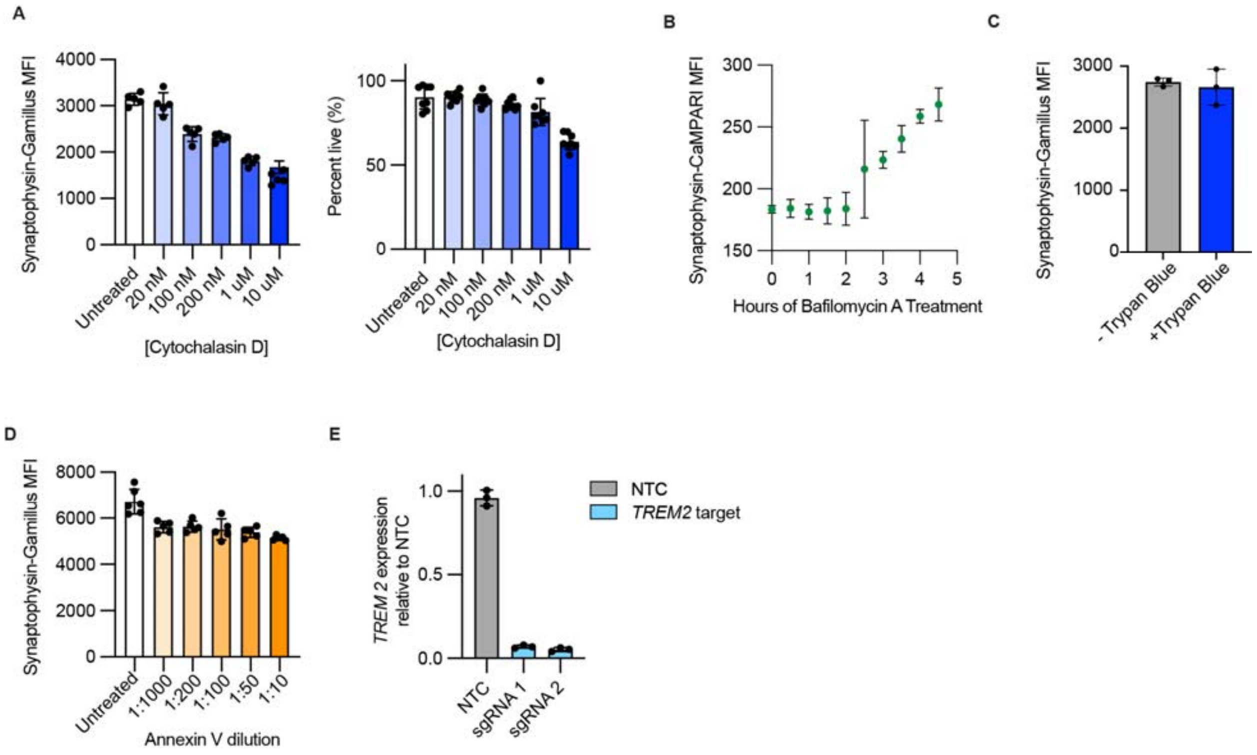

Supplemental Figure 3. Characterization of synaptic pruning assay with pharmacological and genetic perturbations. A. (Left) Uptake of synaptic material by iTF-Microglia measured by flow cytometry after treating cocultures with increasing concentrations of Cytochalasin D (N = 5 wells, bars represent mean +/- standard deviation). (Right) Percent live cells based on nuclear TO-PRO-3 intensity with increasing concentrations of cytochalasin D (N = 4 fields of view from 2 wells, bars represent mean +/- standard deviation). B. Uptake of synaptic material by iTF-Microglia measured by flow cytometry after treating with Bafilomycin A for increasing incubation times. C. Uptake of synaptic material by iTF-Microglia measured by flow cytometry with and without Trypan Blue quenching. D. Uptake of synaptic material by iTF-Microglia measured by flow cytometry after treating cocultures with decreasing dilutions of annexin V (N = 5-6 wells, bars represent mean +/- standard deviation). E. *TREM2* mRNA expression in iTF-Microglia with sgRNAs targeting *TREM2* or non-targeting control (NTC) measured by qPCR.

bioRxiv preprint doi: https://doi.org/10.1101/2024.06.01.596962; this version posted November 17, 2024. The copyright holder for this preprint (which was not certified by peer review) is the author/funder, who has granted bioRxiv a license to display the preprint in perpetuity. It is made available under aCC-BY 4.0 International license.

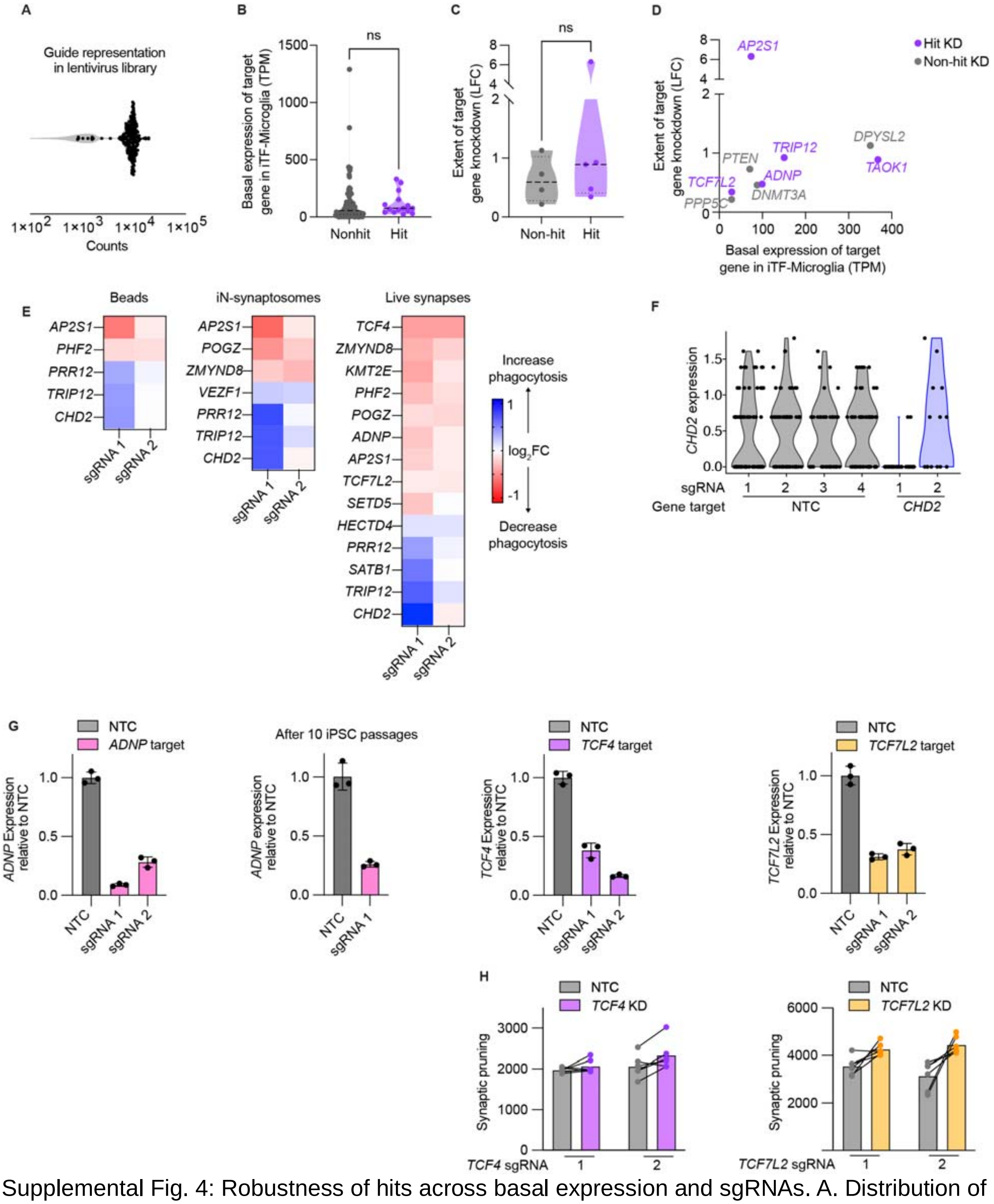

Supplemental Fig. 4: Robustness of hits across basal expression and sgRNAs. A. Distribution of sgRNA representation for CRISPRi-based ASD risk gene library. B. Comparison of iPSC-derived microglia (iTF-Microglia) basal expression of genes that emerged as hits or non-hits across all screens (two-tailed Student's t-test). C. Comparison of knockdown (KD) efficacies based on single-cell transcriptomics dataset for genes that emerged as hits or non-hits across all screens (two-tailed Student's t-test). D. Scatterplot aggregating iTF-Microglia basal expression, extent of KD, and hit classification for target genes. E. Heatmaps for each screen

bioRxiv preprint doi: https://doi.org/10.1101/2024.06.01.596962; this version posted November 17, 2024. The copyright holder for this preprint (which was not certified by peer review) is the author/funder, who has granted bioRxiv a license to display the preprint in perpetuity. It is made available under aCC-BY 4.0 International license.

displaying the phagocytosis phenotype across both guides for genes that emerged as hits. F. *CHD2* expression level from the single-cell transcriptomics dataset for iTF-Microglia with non-targeting controls (NTCs) and iTF-Microglia with sgRNAs targeting *CHD2* (violin plot area represents the density curve). G. *ADNP, TCF4, TCF7L2* mRNA expression in iTF-Microglia with sgRNAs targeting those genes or NTC measured by qPCR. H. Synaptic pruning for iTF-Microglia with individual guides targeting *TCF4* and *TCF7L2*.

bioRxiv preprint doi: https://doi.org/10.1101/2024.06.01.596962; this version posted November 17, 2024. The copyright holder for this preprint (which was not certified by peer review) is the author/funder, who has granted bioRxiv a license to display the preprint in perpetuity. It is made available under aCC-BY 4.0 International license.

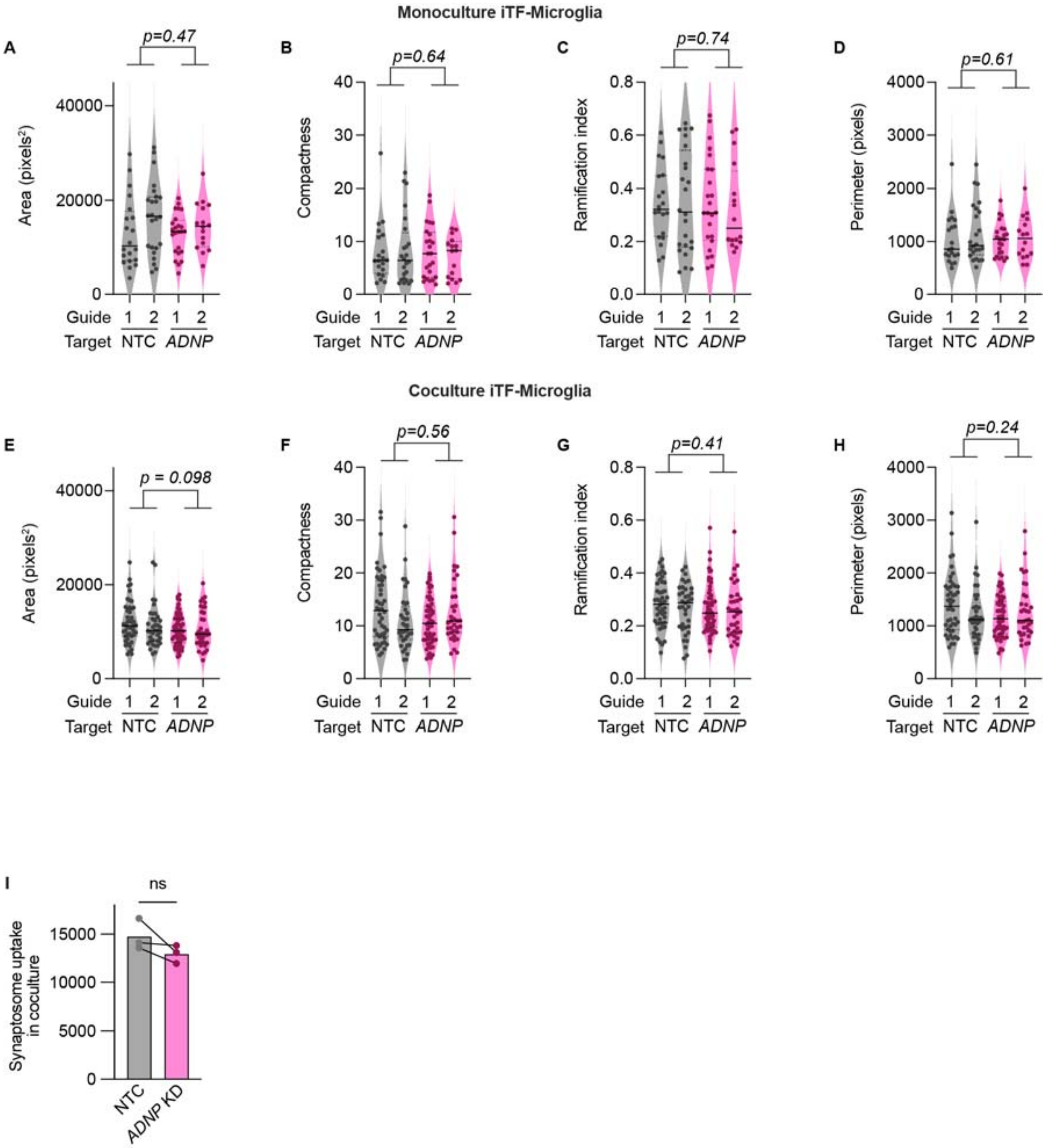

Supplemental Fig. 5: iPSC-derived microglia (iTF-Microglia) morphology and uptake in monoculture and coculture. A-D. Single cell morphology metrics for iTF-Microglia with *ADNP* KD or NTC in monoculture (N = 16-24 single cells, violin plot area represents the density curve, median and quartiles noted as bold and dashed lines respectively, two-way ANOVA). E-H. Single cell morphology metrics for iTF-Microglia with *ADNP* KD or NTC in coculture (N = 35-59 single cells, violin plot area represents the density curve, median and quartiles noted as bold and dashed lines respectively, significance based on NTC vs ADNP factor computed by two-way ANOVA). I. Uptake of iNeuron-derived synaptosomes by iTF-Microglia in coculture measured by flow cytometry (n = 3 wells, bars represent mean +/- standard deviation, paired t-test).

bioRxiv preprint doi: https://doi.org/10.1101/2024.06.01.596962; this version posted November 17, 2024. The copyright holder for this preprint (which was not certified by peer review) is the author/funder, who has granted bioRxiv a license to display the preprint in perpetuity. It is made available under aCC-BY 4.0 International license.

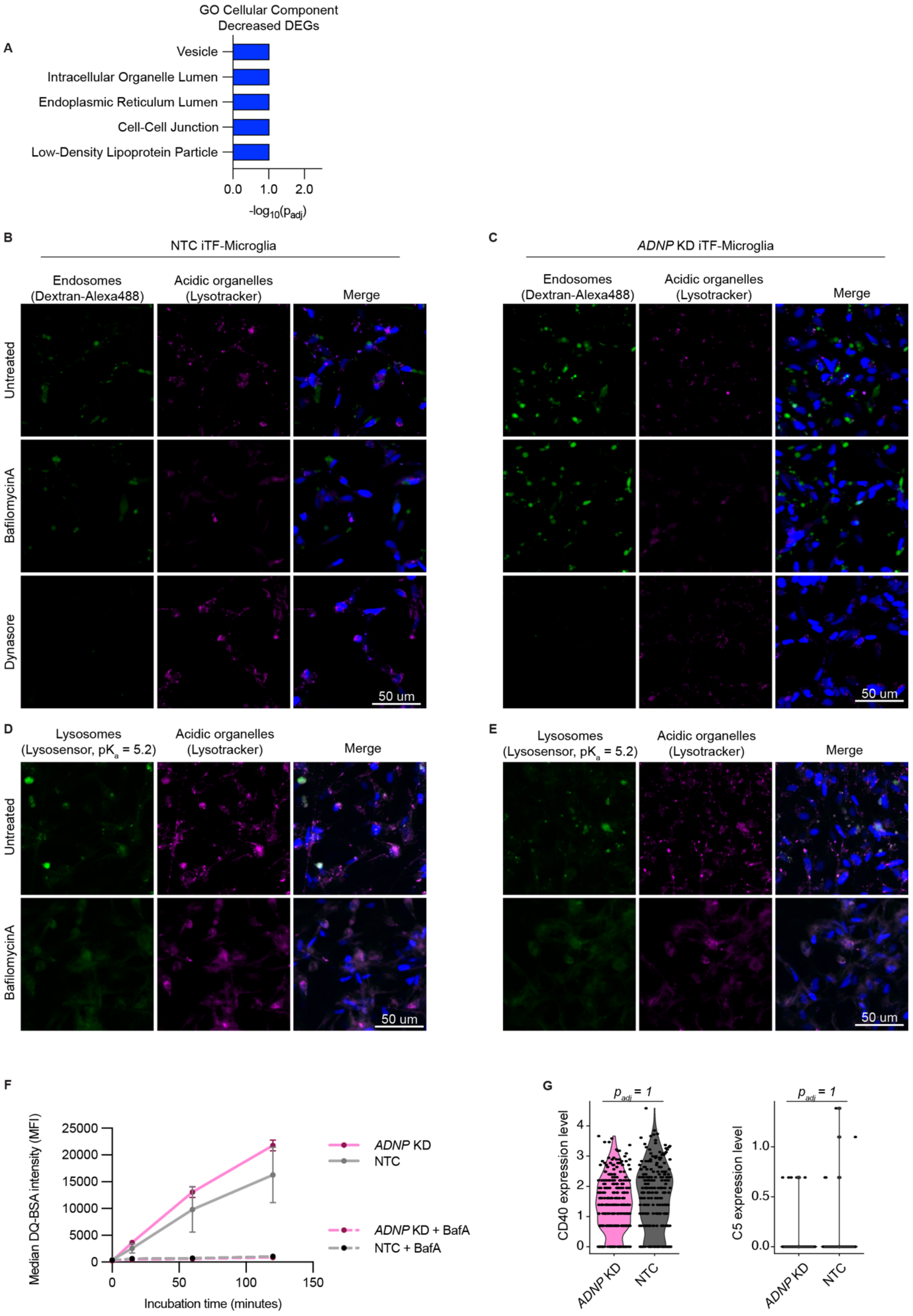

bioRxiv preprint doi: https://doi.org/10.1101/2024.06.01.596962; this version posted November 17, 2024. The copyright holder for this preprint (which was not certified by peer review) is the author/funder, who has granted bioRxiv a license to display the preprint in perpetuity. It is made available under aCC-BY 4.0 International license.

Supplemental Fig. 6: Endolysosomal characterization. A. GO Cellular Component enrichment analysis for downregulated differentially expressed genes for iTF-Microglia with *ADNP* knockdown (KD). B-C. Representative immunofluorescence micrographs of iTF-Microglia with non-targeting control (NTC) (A) or *ADNP* KD (B) incubated with Dextran-Alexa488 (green) and Lysotracker Deep Red (magenta) for 30 minutes with no treatment, BafilomycinA treatment, or Dynasore treatment. D-E. Representative immunofluorescence micrographs of iTF-Microglia with NTC (D) or *ADNP* KD (E) incubated with Lysosensor Green (green) and Lysotracker Deep Red (magenta) for 30 minutes with no treatment or BafilomycinA treatment. F. DQ-BSA fluorescence in iTF-Microglia with NTC (gray lines) or *ADNP* KD (pink lines) with increasing incubation times and BafilomycinA treatment (dashed lines) (N = 3 wells, bars represent mean +/- standard deviation, 2 way ANOVA. G. Expression level from the single-cell transcriptomics dataset for secreted proteins identified by the cytokine array to have increased abundance in iTF-Microglia with *ADNP* KD (violin plot area represents the density curve, two-tailed Student's t-test).

bioRxiv preprint doi: https://doi.org/10.1101/2024.06.01.596962; this version posted November 17, 2024. The copyright holder for this preprint (which was not certified by peer review) is the author/funder, who has granted bioRxiv a license to display the preprint in perpetuity. It is made available under aCC-BY 4.0 International license.

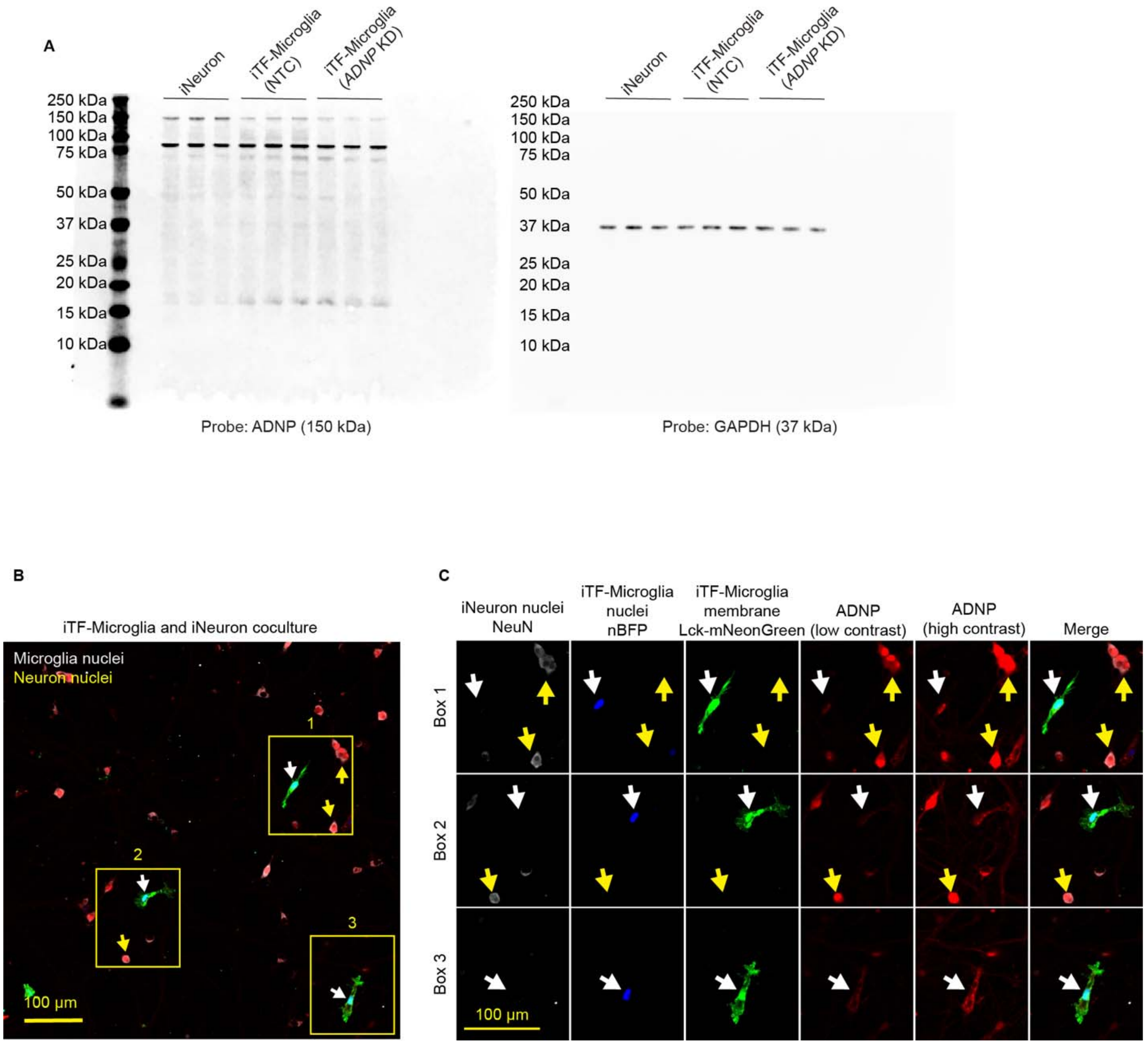

Supplemental Fig. 7: ADNP antibody and localization validation. A. Raw western blot images for ADNP stain (left) and GAPDH (right) for iPSC-derived neurons (iNeurons), iTF-Microglia with non-targeting control, and iTF-Microglia with *ADNP* knockdown (N = 3 replicates per cell type). B. Representative micrograph of iTF-Microglia expressing membrane marker, Lck-mNeonGreen (green) and nuclear BFP (blue), and iNeuron coculture stained with NeuN (gray) and ADNP (red). White arrows indicate microglia and yellow arrows indicate neurons that are shown magnified. C. Insets and individual channels visualizing three iTF-Microglia in the coculture and their ADNP expression.
